# Safety of axitinib and sorafenib monotherapy for patients with renal cell carcinoma: a meta-analysis

**DOI:** 10.7555/JBR.32.20170080

**Published:** 2018-01-26

**Authors:** Fei Qin, Hao Yu, Chang-Rong Xu, Hui-Hui Chen, Jian-Ling Bai

**Affiliations:** Department of Biostatistics, School of Public Health, Nanjing Medical University, Nanjing, Jiangsu 211166, China.; Department of Biostatistics, School of Public Health, Nanjing Medical University, Nanjing, Jiangsu 211166, China.; Department of Biostatistics, School of Public Health, Nanjing Medical University, Nanjing, Jiangsu 211166, China.; Department of Biostatistics, School of Public Health, Nanjing Medical University, Nanjing, Jiangsu 211166, China.

**Keywords:** axitinib, sorafenib, safety, renal cell carcinoma, meta-analysis

## Abstract

We sought to investigate safety of axitinib or sorafenib in renal cell carcinoma (RCC) patients and compare toxicity of these two vascular endothelial growth factor receptor inhibitors. Databases of PubMed and Embase were searched. We included phase II and III prospective trials, as well as retrospective studies, in which patients diagnosed with RCC were treated with axitinib or sorafenib monotherapy at a starting dose of 5 mg and 400 mg twice daily, respectively. The overall incidence of high grade hypertension, fatigue, gastrointestinal toxicity and hand-foot syndrome, along with their 95% confidence intervals (CI), were calculated using fixed- or random- effects model according to heterogeneity test results. A total of 26 trials, including 4790 patients, were included in our meta-analysis. Among them, 6 arms were related to axitinib and 22 were associated with sorafenib. The incidences of hypertension (24.9% *vs.* 7.9%), fatigue (8.2% *vs. *6.6%), and gastrointestinal toxicity (17.6% *vs.* 11.3%) were higher in patients receiving axitinib *versus *those receiving sorafenib, while the incidence of hand-foot syndrome was lower in patients receiving axitinib *versus *those receiving sorafenib (9.5% *vs.* 13.3%). In conclusion, axitinib showed noticeably higher risks of toxicity *versus* sorafenib. Close monitoring and effective measures for adverse events are recommended during therapy.

## Introduction

Renal cell carcinoma (RCC) accounts for 2-3% of all malignant diseases in adults worldwide^[1]^. It was surmised that about 63,000 new cases and 14,000 deaths associated with RCC occurred in the USA in 2016^[2]^. Therapeutic options for this chemotherapy-refractory disease have been constantly updated according to availability of targeted drugs over the past few years. Sorafenib and axitinib are two representative drugs targeting vascular endothelial growth factor receptor (VEGFR) which were approved by the US Food and Drug Administration (FDA) in 2005 and 2012, respectively^[3– 28]^. 

Sorafenib is a tyrosine kinase inhibitor (TKI) that targets molecules involved in tumor cell proliferation and angiogenesis, such as VEGFR-2, VEGFR-3, platelet-derived growth factor (PDGF) receptor-b, c-KIT and FLT-3^[10, 29]^. Axitinib, a second generation TKI, is more potent and selective for VEGFR 1-3^[30]^. The efficacy of axitinib and sorafenib have been demonstrated and compared in two phase III clinical trials^[31]^; however, the result of safety still remains to be defined given the limited sample size and follow-up time. This meta-analysis included available studies of axitinib and sorafenib monotherapy for patients with RCC, and collected safety related data. In this study, we aimed to compare safety and toxicity of axitinib and sorafenib so as to provide evidence for clinical and policy decision-making. 

## Patients and methods

### Search criteria

Trials meeting the following criteria were enrolled: patients were diagnosed with cytologically or pathologically proven advanced/metastatic RCC. Therapy in either arm must be axitinib or sorafenib monotherapy at a starting dosage of 5 mg and 400 mg twice daily, respectively. Prior anticancer therapies including radiotherapy, nephrectomy, interferons and interleukins were permitted. Toxicity data were recorded according to version II or III of the Common Terminology Criteria for Adverse Events (CTCAE) of National Cancer Institute. Trials including concomitant interventions were excluded.

### Search strategy

Databases of PubMed and Embase were reviewed with the following terms: ('sorafenib' OR 'axitinib') AND ('renal cell carcinoma'). Studies which were conducted on or before October 2016 and published only in English were included. This study not only focused on phase II and III clinical trials, but also some retrospective studies, in which axitinib or sorafenib monotherapy was implemented. Phase I trials were not considered given multiple dose levels and limited number of cases. The latest one was adopted if more than one article was found with the same trial. To guarantee that we did not miss any eligible study, related articles from reference list of each study were also retrieved. Further scanning was conducted to determine whether the study was suitable for final analysis.

### Data extraction

Two investigators assessed the eligibility of all the articles independently. The trials were identified through the first author and the year of publication, and divergences were resolved by consensus to ensure the accuracy. Then, trial phase, the number of treated patients, the type and dosage of drugs used in the experimental and control arm, median age and proportion of the male gender were extracted. Toxicity data (grade 3/4 adverse events) recorded in the eligible studies were retrieved, extracted, reorganized and assessed, respectively.

### Statistical methods

For each study, the rate of patients with hypertension, fatigue, diarrhea, decreased appetite, nausea, vomiting and hand-foot syndrome, as well as their 95% confidence intervals (CI), were calculated. To test statistical heterogeneity between studies, the Cochran's Q test was performed. If *P*_heterogeneity_<0.1 or *I*^2^>50%, heterogeneity would be considered to be statistically significant and then data was analyzed through random effects model. Otherwise, a fixed- effects model was applied. Publication bias was estimated using Egger test. Sensitivity analysis was conducted by removing one trial each time to assess the robustness of the finding. Statistical analysis and forest plots were performed using the Comprehensive Meta Analysis version 2 software (Biostat, Englewood, NJ, USA). 

## Results

### Study selection

**Fig.1:**
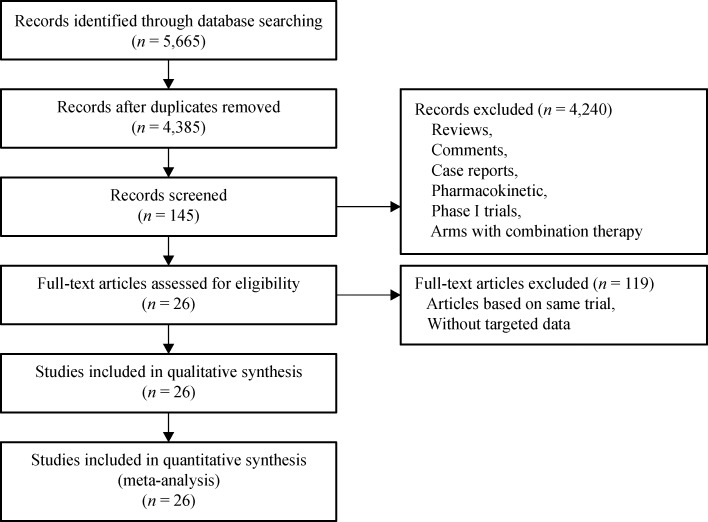
Flowchart of study selection procedure

A total of 1,232 articles on axitinib and 4,433 articles on sorafenib were identified initially from the database and both first and second line treatments were enrolled. Among these, 1,280 were found to be duplicated. After reviewing titles and abstracts, 4,240 subjects were excluded because they were review articles, comments, case reports, pharmacokinetic research or early phase studies (***Fig. 1***). Afterwards, the remaining 145 papers were retrieved for precise browse. Moreover, 119 of the 145 articles were excluded because their results originated from the same patient population in the same trial. Finally, a total of 26 studies were included in this meta-analysis. 

### Study characteristics

Among the trials, a total of 15 trials had only a single arm with axitinib (4 trials) or sorafenib (11 trials). Different kinds of comparators, such as placebo^[9]^, IFN-α-2a^[12]^, temsirolimus^[13]^, and sunitinib^[17, 26]^, were observed in the remaining 11 trials. In two phase III trials^[3– 4]^, axitinib and sorafenib arms were used in the same trial, which resulted in the number of arms exceeding the number of trials in our final analysis. Two phase III trials and four phase II trials regarding axitinib were adopted finally, and the number for sorafenib in each phase reached 6 and 9, respectively. In addition, three retrospective studies and four articles lack of information concerning phase were also enrolled. Their baseline characteristics are listed in ***Table 1***. The number of patients diagnosed with RCC contained in this meta-analysis reached 4,790 and most of them had received previous therapy like cytokine or nephrectomy. Almost all the patients were over 18 years old, with the median age ranging from 52 to 67 years. A significantly higher proportion of the males were observed in each trial, compared with the females. In the arms of patients treated with axitinib, dose escalation was allowed universally, which was nearly reverse in arms of sorafenib except a few trials^[12, 16, 22, 27]^. 

**Tab.1:** Characterisics of trials included in the meta-analysis

Study	Phase	Prior therapy	Age [median(range)]	Male	Dose escalation	Treatment arms	Patients included
Motzer et al. 2013[ 3]	3	Sunitinib, bevacizumab plus interferon alfa, temsirolimus, cytokines	61(20-82) 61(22-80)	265(73%) 258(71%)	Yes No	Axitinib 5 mg b.i.d Sorafenib 400 mg b.i.d	359 355
Hutson et al. 2013[ 4]	3	None	58(23-83) 58(20-77)	134(70%) 74(77%)	Yes No	Axitinib 5 mg b.i.d Sorafenib 400 mg b.i.d	189 96
Rini et al. 2009[ 5]	2	Sorafenib	60(35-77)	42(67.7%)	Yes	Axitinib 5 mg b.i.d	62
Eto et al. 2014[ 6]	2	Cytokine	63(34-80)	44(69%)	Yes	Axitinib 5 mg b.i.d	64
Rixe et al. 2007[ 7]	2	Cytokine	59(35-85)	40(77%)	Yes	Axitinib 5 mg b.i.d	52
Rini et al. 2013[ 8]	2	None	62(28-87)	143(67%)	Yes	Axitinib 5 mg b.i.d	213
Escudier et al. 2009[ 9]	3	Cytokine	58 (19–86) 59 (29–84)	315 (70% 340 (75%)	No	Sorafenib 400 mg b.i.d Placebo	452 451
Ratain et al. 2006[ 10]	2	Cytokine	58(23-83)	149(74%)	No	Sorafenib 400 mg b.i.d	202
Naito et al. 2011[ 11]	2	Cytokine	63(30-83)	100(77.5%)	No	Sorafenib 400 mg b.i.d	131
Escudier et al. 2009[ 12]	2	None	62(34-78) 62.5(18-80)	65(67%) 52(56.5%)	Yes	Sorafenib 400 mg b.i.d IFN-α-2a 9 million U 3 times weekly	97 90
Hutson et al. 2014[ 13]	3	Sunitinib	61(21-80) 60(19-82)	192(24%) 193(25%)	No	Sorafenib 400 mg b.i.d Temsirolimus 25 mg once weekly	252 249
Suzuki et al. 2014[ 14]	Retrospective	Cytokine	67(31-84)	83(74.8%)	No	Sorafenib 400 mg b.i.d	110
Tafreshi et al. 2014[ 15]	NR	Sunitinib, Temsirolimus, Pazopanib	60(34-83)	35(75%)	No	Sorafenib 400 mg b.i.d	47
Garcia et al. 2010[ 16]	2	Bevacizumab, Sunitinib	64(49-79)	34(72%)	Yes	Sorafenib 400 mg b.i.d	47
Zhao et al. 2013[ 17]	Retrospective	None	57(46-67) 52(41-62)	18 15	No	Sorafenib 400 mg b.i.d Sunitinib 50 mg daily	20 23
Beck et al. 2011[ 18]	NR	Cytokine	62(18-84)	858(75%)	No	Sorafenib 400 mg b.i.d	1,145
Procopio et al. 2011[ 19]	2	None	62(52-69) 64(57-69)	43(69%) 52(79%)	No	Sorafenib 400 mg b.i.d Sorafenib plus IL-2	62 66
Motzer et al. 2013[ 20]	3	VEGF-targeted, rapamycin-targeted	59(23-85) 59(23-83)	189(74%) 185(71%)	No	Sorafenib 400 mg b.i.d Tivozanib 1.5 mg once daily	257 259
Jonasch et al. 2010[ 21]	2	None	62.4(45-83) 60.7(43-81)	32(80%) 29(72.5%)	No	sorafenib 400 mg b.i.d Sorafenib+ IFN	40 40
Amato et al. 2012[ 22]	2	Cytokine	62.5(42-78)	37(84%)	Yes	Sorafenib 400 mg b.i.d	45
Laber et al. 2009[ 23]	NR	Cytokine	64(55-82)	10(71.4%)	No	Sorafenib 400 mg b.i.d	14
Motzer et al. 2014[ 24]	3	Cytokine, VEGF-targeted, mTOR inhibitor	62(18-81) 61(29-89)	219(77%) 213(75%)	No	Sorafenib 400 mg b.i.d Dovitinib	284 280
Yang et al. 2012[ 25]	NR	Cytokine	(18-80)	21(70%)	No	Sorafenib 400 mg b.i.d	30
Park et al. 2012[ 26]	Retrospective	None	62(26-85) 56.5(17-86)	35(71%) 161(73%)	No	Sorafenib 400 mg b.i.d Sunitinib 50 mg once daily	49 220
Wang et al. 2014[ 27]	2	None	53(24-81)	33(80%)	Yes	Sorafenib 400, 600, 800 mg b.i.d	41
Hainsworth et al. 2013[ 28]	2	Bevacizumab, Sunitinib	62(44-86)	52(69%)	No	Sorafenib 400 mg b.i.d	75

NR, not reported; IFN-α-2a, interferon alfa-2a; IL-2, interleukin-2; IFN, interferon.

### Incidence of adverse events

#### Hypertension

**Fig.2:**
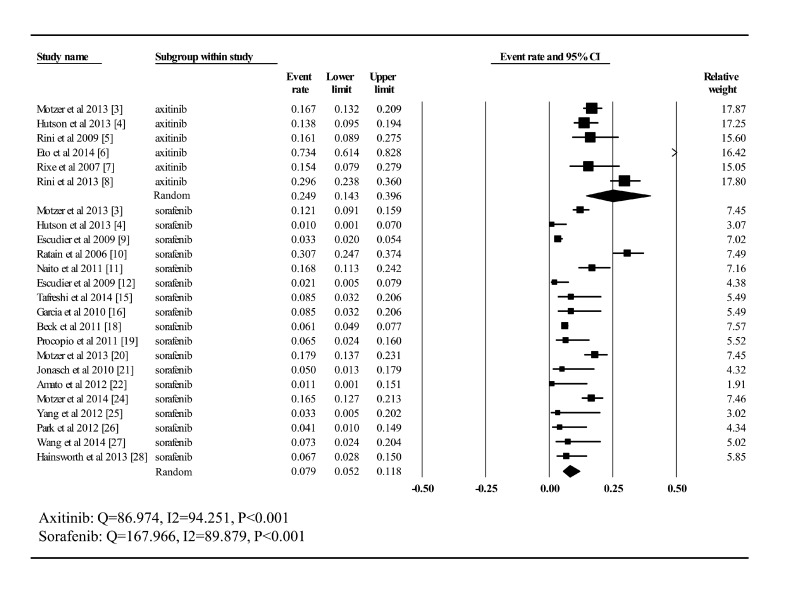
Incidence of high-grade hypertension to axitinib and sorafenib

**Fig.3:**
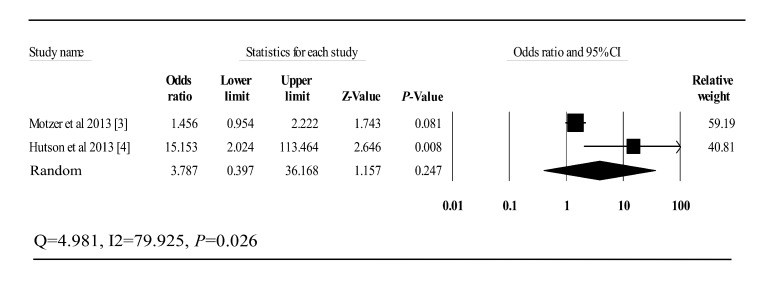
Odds ratio of axitinib and sorafenib for high-grade hypertension in two phase III trials

All the six clinical trials related to axitinib, including a total of 939 patients, had data of high grade hypertension available for analysis (***Fig. 2***). The incidence remained stable with slight fluctuation at around 2% among most trials except a Japanese phase II study, which dramatically jumped to 73.4%. As for sorafenib, the number of trials providing data on high-grade hypertension was 18 and the proportion ranged from 0% to 30.7%. The unique trial reporting no hypertension patients was an intrapatient dose escalation study^[22]^. The summary incidence of high-grade hypertension in 3455 patients receiving sorafenib was estimated as 7.9% (CI: 5.2%–11.8%), compared with 24.9% (CI: 14.3%–39.6%) for axitinib, after using the random-effects model for analysis (*Q*= 86.974, *I*^2^= 94.251, *P*<0.001; *Q*= 167.966, *I*^2^= 89.879, *P*<0.001). In addition, two phase III trials which involve both axitinib and sorafenib monotherapy arm were found during selection process. Thus, an extra analysis was conducted for these two studies and odds ratio (95% CI) for high grade hypertension was 3.787 (0.397–36.168) (***Fig. 3***). 

#### Fatigue

**Fig.4:**
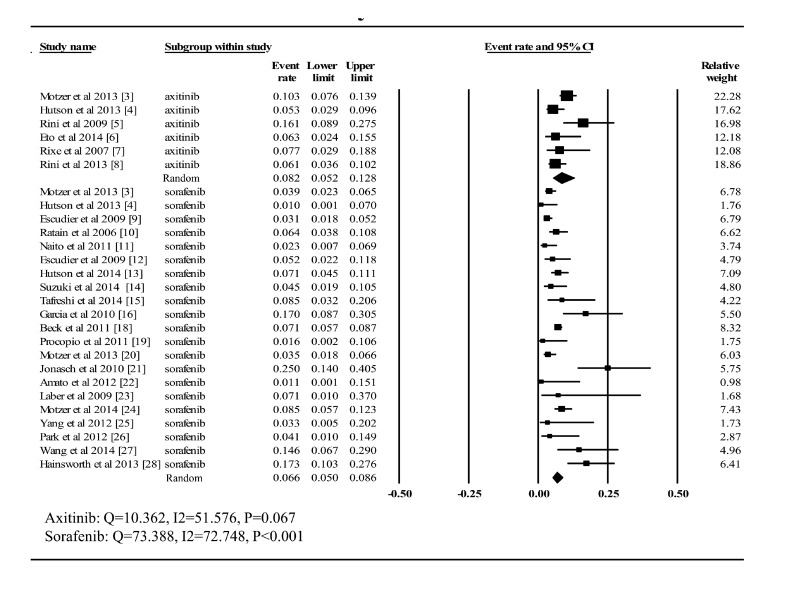
Incidence of high-grade fatigue to axitinib and sorafenib

As shown in ***Fig. 4***, information regarding high-grade fatigue was available in all six trials associated with axitinib and the incidence fluctuated between 5.3% and 16.1%. Taking it into consideration that heterogeneity had been proved to be statistically significant (*Q*= 10.326, *I*^2^= 51.576, *P* = 0.067), the random-effects model was adopted to compute the summary proportion (8.2%, CI: 5.2%-12.8%). Among the trials of patients treated with sorafenib, only one study lacked high-grade fatigue data^[17]^. The largest incidence (25%) was revealed in a phase II study comparing sorafenib monotherapy with combination therapy with sorafenib and low-dose interferon alfa. Similarly, forest plot was performed using the random-effects model (*Q*= 73.388, *I*^2^= 72.748, *P*<0.001), and the summary rate (6.6%; CI: 5.0%–8.6%) was slightly lower than that hypertension. 

#### Gastrointestinal toxicity

According to data extracted in our meta-analysis, gastrointestinal toxicity was universal in almost every trial. The summary incidence of high-grade diarrhea, decreased appetite, nausea and vomiting during treatment with axitinib or sorafenib is presented in ***Table 2***, and the possibility for patients diagnosed with these adverse events after receiving axitinib was obviously larger than that in sorafenib arms. 

**Tab.2:** Summary incidence of gastrointestinal toxicity

	Axitinib (summary incidence)	Sorafenib (summary incidence)	
Diarrhea	9.8% (CI: 8.1%-12.0%)	5.9% (CI: 4.5%-7.8%)	
Decreased appetite	3.5% (CI: 2.4%-4.9%)	2.8% (CI: 2.2%-3.4%)	
Nausea	2.3% (CI: 1.4%-3.6%)	1.4% (CI: 0.8%-2.4%)	
Vomiting	2.0% (CI: 1.1%-3.3%)	1.2% (CI: 0.9%-1.8%)	

#### Hand-foot syndrome

**Fig.5:**
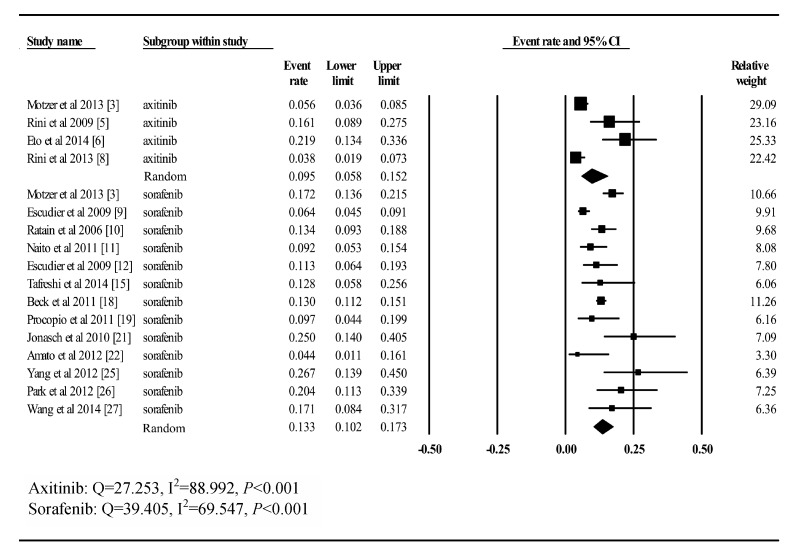
Incidence of high-grade hand-foot syndrome to axitinib and sorafenib

A total of 16 trials, including 698 patients treated with axitinib and 2696 patients treated with sorafenib, provided toxicity data on high-grade hand-foot syndrome in our meta-analysis. Using the random-effects model (*Q*= 27.253, *I*^2^= 88.992, *P*<0.001; *Q*= 39.405, *I*^2^= 69.547, *P*<0.001), the incidences in summary were 9.5% (CI: 5.8%–15.2%) for sorafenib and 13.3% (CI: 10.2%–17.3%) for axitinib (***Fig. 5***). 

#### Publication bias

Publication bias was not detected for the incidence of each high grade safety effect except for decreased appetite in the sorafenib group (Egger's test: *P* = 0.012). 

## Sensitivity analysis

Sensitivity analysis indicated that for all the adverse events reported in this meta-analysis, no trial interrupted the robustness of the whole research seriously exept the trial from Eto *et al*.^[6]^ for the occurence of hypertension. The summary incidence went down from 0.249 to 0.182 after removing this trial. 

## Discussion

The toxicity (e.g. hypertension, gastrointestinal effects and hand-foot syndrome) related to VEGFR inhibitors has been previously reported in several systematic reviews^[32– 33]^. However, the results from most previous reviews evaluated safety effects of combination therapy. Therefore, we conducted a meta-analysis here, where only studies with axitinib or sorafenib monotherapy were enrolled. 

Axitinib has been demonstrated to prolong progression free survival (PFS) (axitinib vs. sorafenib, median PFS 6.7 *vs.* 4.7 months) in a phase III study^[31]^. However, its toxicity in causing hypertension should not been ignored. In this meta analysis, the incidence of high grade hypertension for patients receiving axitinib tripled compared to that for sorafenib (24.9% *vs.* 7.9%). Mostly, hypertension originates from anti-VEGF activities^[32]^. VEGF plays an essential role in promoting endothelial cell proliferation, as well as its survival. Conversely, once VEGF is inhibited, peripheral resistance will trend to ascend given endothelial cell damage and dysfunction^[34– 35]^. Besides, another mechanism concerning the occurrence of hypertension is considered to be attenuated nitric oxide (NO) production on the surface of different types of vessels^[36]^. Actually, NO is a vasodilator, and the decrease of NO synthesis may promote vasoconstriction, which will then lead to increased blood pressure. Interestingly, the results from a pharmacokinetic and pharmacodynamic analysis revealed that the increase of diastolic blood pressure can predict favorable PFS and overall survival^[37]^. Moreover, treatment of hypertension during axitinib experiment would not undermine the efficacy of drugs. Though the association between hypertension and efficacy has been revealed, further research about how they interact with each other still remains to be done. 

In addition to hypertension, fatigue and gastrointestinal toxicity like diarrhea, decreased appetite, nausea and vomiting were also common events observed in studies of VEGFR inhibitors. Generally, therapy was generally not suspended if the above events occurred. With the help of dietary intervention or combination therapy, symptoms can be controlled and mitigated. For elderly patients, if high grade diarrhea or vomiting is not controlled well, worse effects like dehydration may occur^[38]^. Furthermore, it has been reported that treatment-related diarrhea can prolong the duration of multikinase therapy, reduce the mobility and compromise quality of life^[39]^. As a result, clinical guidelines for managing tumor treatment-related gastrointestinal adverse events should be well conducted. 

It is reported that patients receiving axitinib were less likely to suffer from hand food skin reaction (HFSR), compared to patients with sorafenib (9.5% *vs.* 13.3%). Early in 2007, HFSR was found to be the most evident dermatologic adverse event in patients treated with sunitinib and sorafenib^[40– 41]^. HFSR was also observed in axitinib treated patients in recent years. The reason for the high incidence of HFSR in sorafenib patients may be that simultaneous inhibition of VEGFR and PDGFR will interrupt normal vascularity, which is indispensable during the repair of fibroblasts and endothelial cells^[42– 43]^. Interestingly, when VEGFR or PDGFR is separately inhibited with imatinib or some molecules antibodies^[44– 45]^, HFSR is not common. However, axitinib, a specific VEGFR inhibitor, is reported to have comparable incidence here. Actually, the mechanism for this is still not clear, and thus the potential impact of axitinib on PDGFR and VEGFR was originally underestimated. In addition, hypertension due to axitinib may result in vasoconstriction in the sensitive skin^[46]^. Though HFSR seems to be general for patients treated with sorafenib or axitinib, some precautions, such as removing hyperkeratotic areas prophylactically, wearing soft shoes, avoiding exercises prone to increase friction on the palms and soles^[47]^ and use of urea^[48]^, may be undertaken. 

It is important to mention that a couple of limitations still existed in this meta-analysis. First, most studies involved were conducted in institutions from different countries. As a result, potential bias may exist in reporting adverse events. Secondly, we included both prospective and retrospective trials in this analysis, and data was collected during various periods of the study. Moreover, the requirements for dose escalation are not consistent between trials. All of these would increase heterogeneity among the included studies. Thirdly, studies here were conducted in patients only with adequate organ function. Therefore, incidence and its 95% CI calculated in the article may not be applicable to overall population.

In conclusion, axitinib showed noticeably higher risks of toxicity *versus* sorafenib. Our results indicate that strict monitoring and effective management should be conducted to prevent severe safety effects during therapy with sorafenib and axitinib. 
